# A Microdevice Platform Recapitulating Hypoxic Tumor Microenvironments

**DOI:** 10.1038/s41598-017-15583-3

**Published:** 2017-11-09

**Authors:** Yuta Ando, Hoang P. Ta, Daniel P. Yen, Sang-Sin Lee, Sneha Raola, Keyue Shen

**Affiliations:** 1 Department of Biomedical Engineering, Viterbi School of Engineering, University of Southern California, Los Angeles, CA 90089 USA; 2 Norris Comprehensive Cancer Center, Keck School of Medicine, University of Southern California, Los Angeles, CA 90033 USA; 3 Department of Stem Cell Biology and Regenerative Medicine, Keck School of Medicine, University of Southern California, Los Angeles, CA 90033 USA

## Abstract

Hypoxia plays a central role in cancer progression and resistance to therapy. We have engineered a microdevice platform to recapitulate the intratumor oxygen gradients that drive the heterogeneous hypoxic landscapes in solid tumors. Our design features a “tumor section”-like culture by incorporating a cell layer between two diffusion barriers, where an oxygen gradient is established by cellular metabolism and physical constraints. We confirmed the oxygen gradient by numerical simulation and imaging-based oxygen sensor measurement. We also demonstrated spatially-resolved hypoxic signaling in cancer cells through immunostaining, gene expression assay, and hypoxia-targeted drug treatment. Our platform can accurately generate and control oxygen gradients, eliminates complex microfluidic handling, allows for incorporation of additional tumor components, and is compatible with high-content imaging and high-throughput applications. It is well suited for understanding hypoxia-mediated mechanisms in cancer disease and other biological processes, and discovery of new therapeutics.

## Introduction

Cancer remains one of the leading causes of death despite of the vast investment and efforts in research and drug development. Over 1.68 million new cancer cases and 0.6 million cancer deaths are projected to occur in the United States alone in 2017^1^. Resistance towards conventional chemo- and radio-therapies as well as the fast-growing immunotherapies presents a significant challenge in cancer treatments, particularly in solid tumors^2,3^. The tumor microenvironment (TME) consists of complex cellular and molecular interactions that regulate the progression and therapeutic response of tumors^4^. Hypoxia, the condition of oxygen deficiency, is a central player in the TME and cancer progression^5,6^. Notably, degrees of hypoxia in solid tumors are very heterogeneous and can range from 0.5–2% oxygen saturation compared to 4–7% in healthy tissues and 21% in atmospheric air^7,8^. Different degrees of hypoxia induce varying levels of metabolic adaptation, extracellular matrix (ECM) remodeling, epithelial-mesenchymal transition (EMT), angiogenesis, pH regulation, and immune suppression^9,10^. It also promotes cancer stem-like cell (CSC) phenotypes, adding to tumor heterogeneity and therapy resistance^11^. Recapitulating *in vivo* hypoxic conditions will therefore facilitate the screening and development of new therapeutics^12^.

Considerable efforts have been made to establish hypoxic tumor models that can be analyzed with ease and reproducibility. *In vivo* models provide naturally formed^13^ or induced^14^ hypoxia. However, these models typically involve significant individual variabilities, high cost, and low throughput^15–17^. They also have limited spatiotemporal and cellular resolutions inherent to most *in vivo* imaging modalities^17^. *In vitro* models can provide a high level of manipulation, specificity, sensitivity, and reproducibility that are difficult to obtain *in vivo* ^16^. Hypoxia can be induced *in vitro* using chemical methods^18^, hypoxia chambers^19^, spheroid cultures^20^, and micro-engineering approaches^21^. Chemical induction of hypoxia can adversely affect signaling pathways other than those regulated by hypoxia^18^. Commercially available hypoxia chambers provide one oxygen concentration at a time, thus limiting its throughput in testing cell responses to different oxygen levels. Moreover, these approaches fail to capture the spatial complexity of oxygen profiles and the resulted crosstalk in a hypoxic tumor^22,23^. Tumor spheroid cultures can induce a hypoxic gradient that histologically resemble avascular tumor nests^24^. However, spheroids are generally incompatible with high-content analysis such as live-cell tracking and spatiotemporally resolved single-cell analysis, which would otherwise require laborious post-processing such as embedding and sectioning, or expensive, deep imaging platforms^25,26^. Engineered 3-dimensional (3D) cultures have also emerged as an alternative method to capture gradients of oxygen and nutrients. For instance, paper-supported 3D cell cultures have been developed to recapitulate gradients in spheroids and tumors, where layers of 2D cultures are stacked to establish the gradients, and disassembled for imaging and analysis^27^. Such methods lack a lateral gradient profile for microscopy, and require additional handling to analyze cells on each layer. Microfluidic platforms have been established to create oxygen gradients on a lateral surface to facilitate microscopic observation^28–32^. However, they often face challenges of high oxygen permeability of fabrication materials, maintenance of an accurate gradient, complicated fabrication processes, and microfluidic design/handling that are challenging to biological research laboratories. Those designs with continuous flow over the cells also prohibits lateral cell-cell communications between gradient zones through soluble mediators^33^. To date, there has not been a user-friendly, scalable *in vitro* hypoxic model that mimics the *in vivo* oxygen gradient and is compatible with high-content imaging and high-throughput applications.

In this study, we take a novel approach to recapitulate a hypoxic gradient within a micropatterned monolayer culture of human cancer cells. Cellular metabolism is combined with micromilled oxygen diffusion barriers to establish a natural hypoxic gradient. Induction of hypoxia in our microdevice is driven by cellular oxygen consumption, similar to the formation of tumor hypoxia due to increased oxygen demand by uncontrollably proliferating cells; therefore, we are able to mimic natural hypoxia induction while eliminating the need for an external source of oxygen control. The platform is integrated with oxygen sensors for real-time, spatially-resolved measurements and is compatible with microscopy-based techniques. It enables high-content, spatially-resolved analyses of cell phenotypic and gene expressions, and further allows for assessment of hypoxia-targeted drugs, as demonstrated in this study using tirapazamine (TPZ). We present the device and platform as a versatile tool for gaining insights into cancer biology and accelerate the development and discovery of new therapeutics.

## Results

### Diffusion barriers create an oxygen gradient in a cell layer

**Figure 1:**
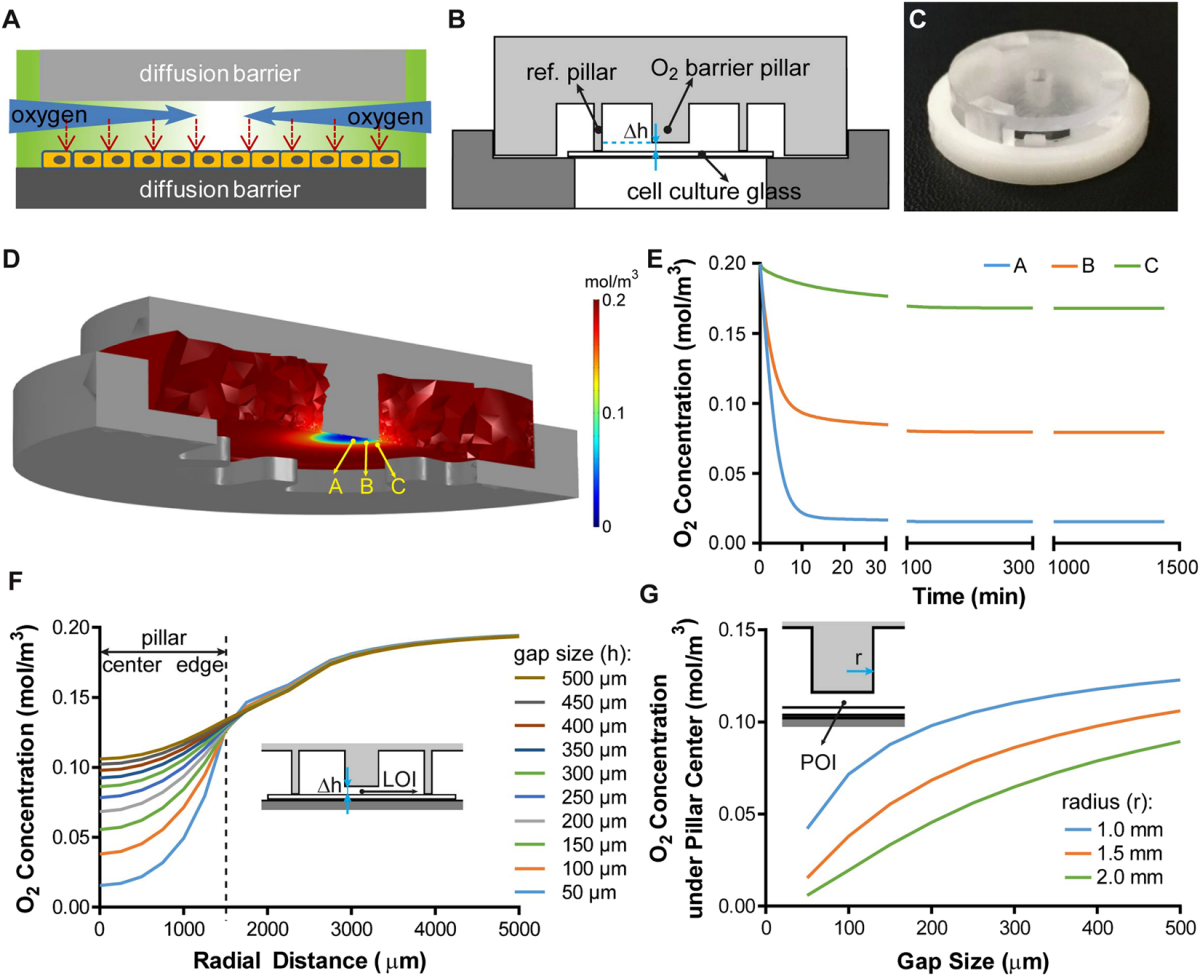
Recapitulation of a gradient of oxygen in a hypoxia microdevice and finite element analysis of expected oxygen diffusion by COMSOL Multiphysics^®^. (**A**) Illustration of the working principle. (**B**) Cross-section side view of the microdevice. (**C**) CNC milled and assembled microdevice. (**D**) Steady state oxygen in the hypoxia microdevice as a result of oxygen barriers and oxygen consumption by a micropatterned cell layer. (**E**) Evolvement of oxygen levels in the microdevice within 1,440 minutes of device assembly with the micropatterned cell monolayer. (**E**) Modulation of the steady state oxygen distribution by the gap size in a hypoxia microdevice. (**F**) Sensitivity of oxygen level at pillar center to gap sizes and radii of the oxygen barrier pillar.

In a 3D tumor mass, a gradient of oxygen or hypoxia is established by the combined effects of cellular metabolism and oxygen diffusion. With the same concept, we introduced a “tumor-section”-like monolayer culture that incorporates “insulated” oxygen boundary conditions on both sides of the monolayer (Fig. 1A). Metabolic consumption and limited passive diffusion of oxygen in the gap between the two barriers will thus result in a gradient of oxygen and hypoxic levels in the monolayer (Fig. 1A). To achieve this theoretical induction of hypoxia, we created a microdevice using a computer numerical control (CNC) micromilling platform with high precision at the microscale^34^. The microdevice consists of (1) a cap structure with a central pillar as an oxygen barrier and three reference pillars that determine the gap size for oxygen diffusion, and (2) a base structure that holds a culture substrate with a cell monolayer (Fig. 1B). In its assembled form, a Delrin® base (white) holds a gas-impermeable glass coverslip with a cell monolayer to form the bottom diffusion barrier and the oxygen consumption layer; a polycarbonate pillar provides the other oxygen diffusion barrier as well as a transparent observation window for microscopy (Fig. 1C). For this study, we have chosen polycarbonate as the cap material due to its low oxygen permeability (polycarbonate: 9.1 × 10^−9^ cm^3^∙cm/(cm^2^∙sec∙atm); polydimethylsiloxane (PDMS, commonly used in soft lithography and microfluidic devices): 4.7 × 10^−6^ cm^3^∙cm/(cm^2^∙sec∙atm))^29,35–37^ and excellent optical transparency^34^.

We next carried out computer simulations with COMSOL Multiphysics^®^ to characterize the spatial and temporal profiles of oxygen expected in the hypoxia device. Figure 1D demonstrates the steady-state distribution of oxygen in a microdevice with a 50 µm diffusion gap. The heat-map of oxygen concentration shows a radial transition of oxygen concentrations from near-zero under the center of the pillar to a normoxic level at the periphery. We selected three points of interest (POIs) immediately above the cell monolayer: at the center (A), at the intermediate region (B) and at the edge (C) in relation to the pillar geometry (Fig. 1D). It was found that oxygen level within the device drops quickly within the first 10 minutes. Within 30 minutes, the oxygen levels are already within 92.8%, 93.3%, and 94.5% of the steady-state level at the locations A, B, and C, respectively. We then examined the influence of the diffusion gap size on the steady-state oxygen profile above the cell layer. This parameter is important for the design of the hypoxia microdevice, as actual oxygen level under the pillar can be influenced by the micromilling accuracy of Δh. As shown in Fig. 1F, the radial oxygen distribution can be fine-tuned with Δh, where smaller gaps result in steeper oxygen gradients under the pillar. Oxygen gradient outside the pillar has little dependence on the gap size, suggesting that the device structure other than the pillar does not hinder oxygen supply from the bulk media and the media-air interface. We further investigated the sensitivity of oxygen concentration to the pillar radii. We simulated the oxygen level at the center in relation to Δh under pillars of different radii (Fig. 1G). A larger radius increases the distance of oxygen diffusion, thus lowering the oxygen concentration in the center. COMSOL^®^ simulation showed that in general, oxygen levels under smaller pillars are more sensitive to the changes in Δh (Fig. 1G). 1.5 mm was chosen as the pillar radius in the study to achieve biologically relevant hypoxic level^38,39^, manageable imaging area for microscopy, and minimal sensitivity to the variation of Δh in micromilling^34^.

### An integrated sensor layer can monitor the oxygen gradient

**Figure 2:**
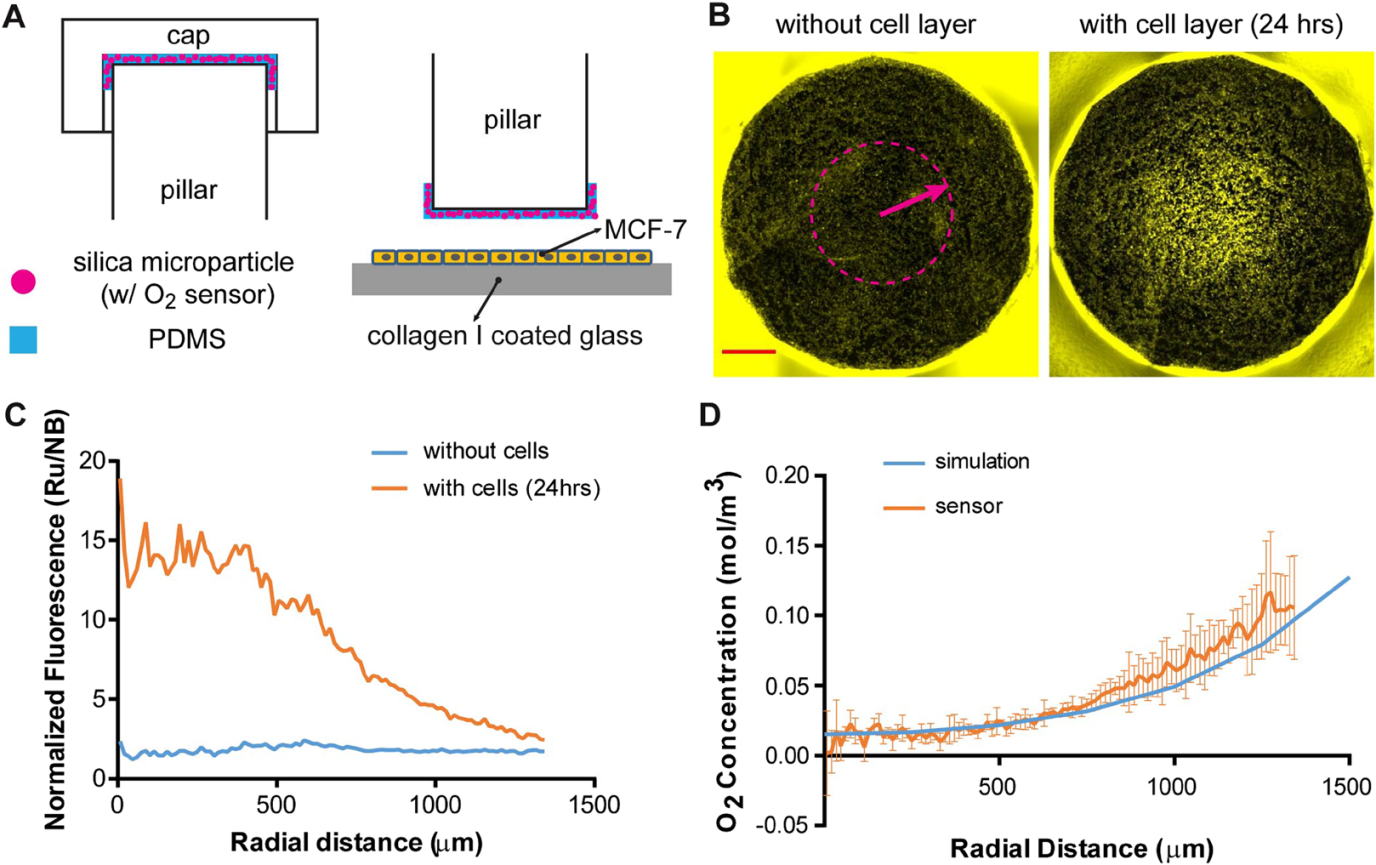
Oxygen levels in the microenvironment measured by an oxygen-sensitive fluorophore. (**A**) Schematics of oxygen sensor layer in the hypoxia device. (**B**) Fluorescent signal from sensor layer without or with the cell layer in the device. Scale bar: 500 µm. (**C**) Normalized fluorescent intensity of ruthenium compound by Nile blue in oxygen sensor particles over radial distance (center to edge) with and without cell layer from the same pillar. (**D**) Derived oxygen concentration under the pillar (orange, N = 3) compared to simulated oxygen concentration (blue) show a good correlation (Pearson’s correlation coefficient r = 0.9458). Error bars: standard deviation (SD).

Next, we performed cell culture experiments and characterized the actual oxygen gradient by embedding a fluorescence-based oxygen sensor in the microdevice. Silica microparticles were absorbed with an oxygen-sensitive luminophore Ru(Ph_2_phen_3_)Cl_2_ (oxygen sensor) mixed with an oxygen-insensitive fluorophore, Nile blue chloride (control)^40^. They were then mixed in PDMS and spread in a thin layer onto the oxygen barrier pillar using a micromilled cap (Fig. 2A, left panel). MCF-7 cells, a breast cancer cell line, were micropatterned on a collagen I coated coverslip in a circular island to mimic the morphology of cancer cell nests in a tumor tissue section^41^, and assembled into the hypoxia microdevice (Fig. 2A, right panel). After 24 hours of cell culture, we obtained images of the silica microparticles in the respective fluorescent channels for the ruthenium compound and Nile blue chloride. Enhanced fluorescent signal from the ruthenium compound was observed near the center of the pillar in a radial distribution profile. In contrast, the same microdevice without the cell monolayer showed a relatively uniform, dim fluorescent signal (under the same imaging settings), which is consistent with the oxygen-quenching property of the ruthenium compound (Fig. 2B). Signal from Nile blue chloride, on the contrary, was insensitive to oxygen concentrations (data not shown). The normalized fluorescent signal (ruthenium by Nile blue, Ru/NB) was plotted against the radial distance without or with the cell layer from a single microdevice (Fig. 2C). Normalized fluorescence intensity was converted to oxygen concentration (N = 3) and compared against the COMSOL prediction, which shows a good match between the two (Pearson’s correlation coefficient r = 0.9458) (Fig. 2D).

### Hypoxic markers are upregulated in the microdevice

**Figure 3:**
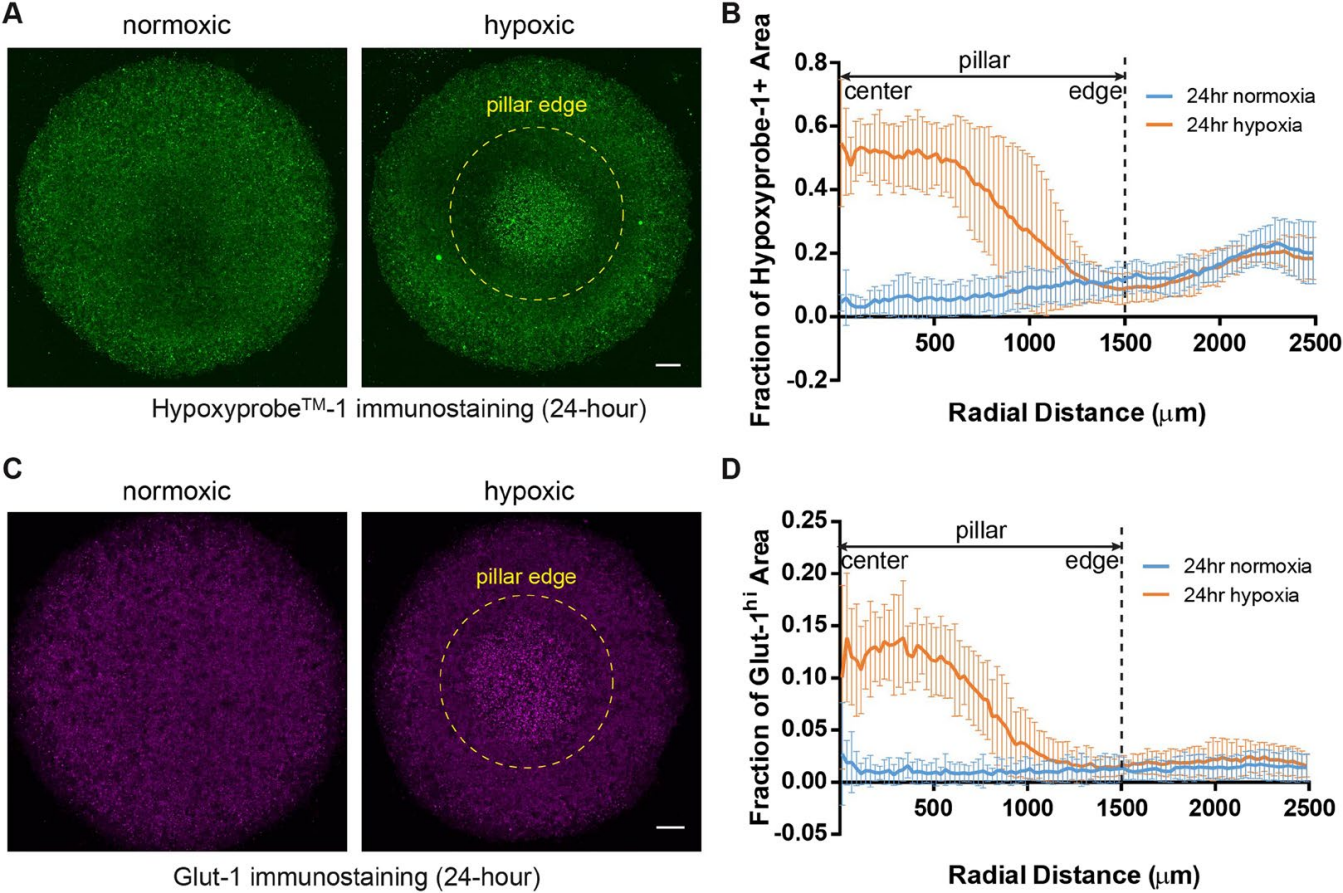
Upregulation of hypoxic markers in microdevice. (**A**) Hypoxyprobe^TM^-1 immunostaining in micropatterned MCF-7 cells under normoxic condition and in hypoxia device after 24 hours of incubation. (**B**) Radial analysis of areal fractions with high Hypoxyprobe^TM^-1 signal (normoxia: N = 4; hypoxia: N = 7). (**C**) Glut-1 immunostaining in micropatterned MCF-7 cells under normoxic condition and in hypoxia device after 24 hours (normoxia: N = 5; hypoxia: N = 8). (**D**) Radial profile of areal fractions with high Glut-1 expression. Scale bars: 500 µm. Error bars: SD.

We next carried out immunofluorescent analysis on hypoxic markers to confirm that cells can create and respond to the oxygen gradient in the microdevice. Pimonidazole (also known as Hypoxyprobe^TM^-1) is a chemical compound that can be reduced in hypoxic cells to form stable covalent adducts with thiol groups in proteins, peptides and amino acids, which can then be detected by immunofluorescent staining^42,43^. Elevated pimonidazole staining was detected under the pillar (Fig. 3A,B). Quantitative analysis (N = 7) showed a signal plateau near the center of the pillar (in ~600 µm radius), with gradual decline to a background level near the edge of the pillar (from 600 to 1,300 µm). The oxygen concentrations corresponding to the two transition points are 0.028 and 0.08 mol/m^3^, respectively, based on the COMSOL Multiphysics^®^ simulation.

Glucose transporter-1 (Glut-1) is a glucose transporter protein that facilitates glucose supply into cells. It has been established as an intrinsic cellular marker for hypoxia and correlated with levels of reduced pimonidazole^44,45^. We immunostained Glut-1 in the MCF-7 cells incubated for 24 hours in the microdevice, and observed distinct Glut-1 upregulation under the pillar (Fig. 3C,D). A high degree of correlation was observed between the radial profiles of Glut-1 and reduced pimonidazole in the hypoxia device (Pearson’s correlation coefficient r = 0.9699), which agrees with previous findings^42^.

### Gene expressions are spatially regulated in the microdevice

**Figure 4:**
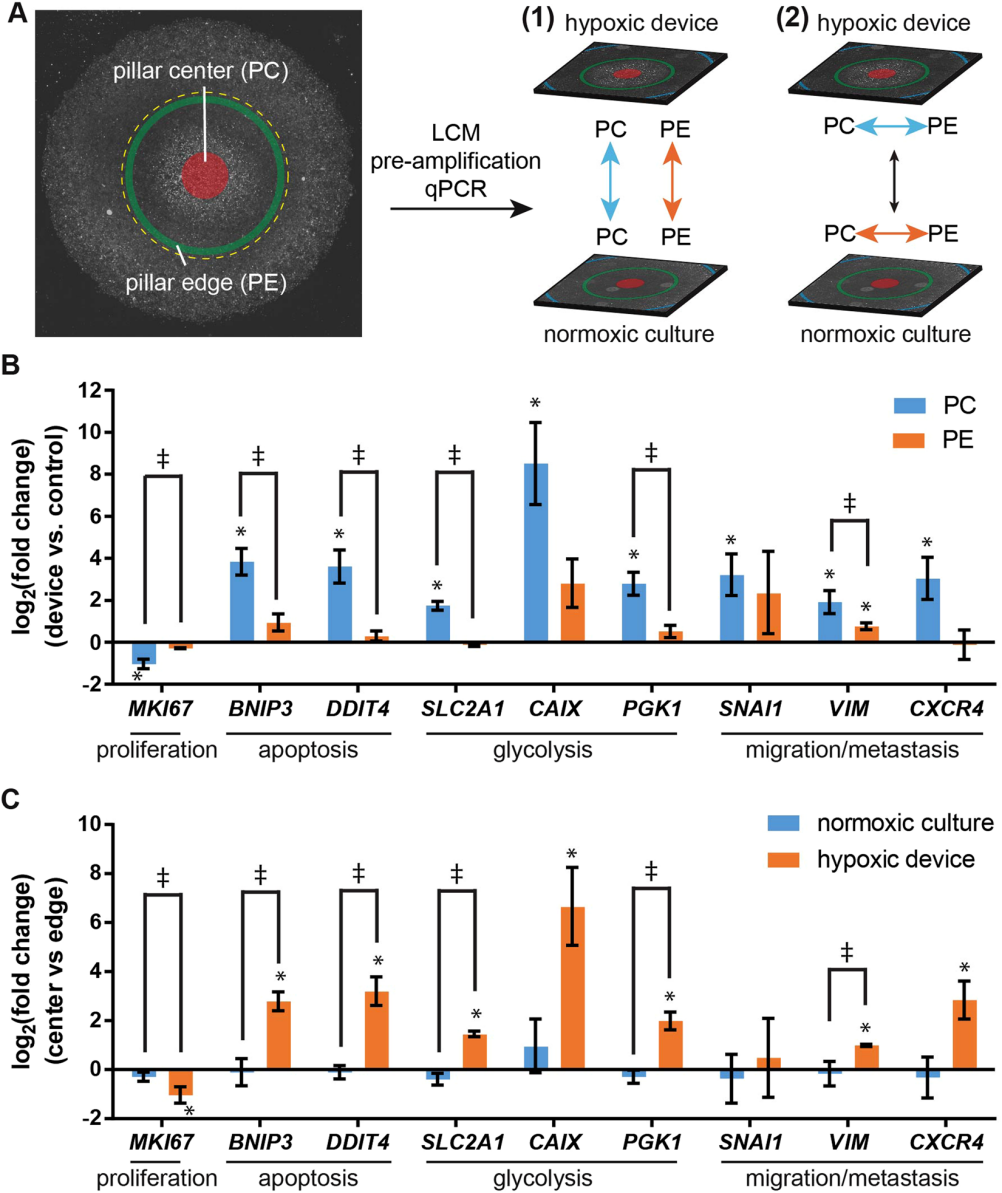
Gene expression analysis in hypoxia device. (**A**) Areal definition for laser capture microdissection in micropatterned MCF-7 cells under hypoxia device and two types comparisons of gene expression (1: hypoxia vs normoxia; 2: center vs edge). PC: pillar center; PE: pillar edge. (**B**) Region-by-region comparison between hypoxic and normoxic samples, and (**C**) In-sample comparison between center and edge areas under hypoxia device, with genes related to proliferation, apoptosis, glycolysis, and migration/metastasis. (**B**,**C**) N = 3. Student’s t-test: *p < 0.05 for significant fold change in gene regulation; ǂp < 0.05 for significant paired difference. All other conditions (non-labeled): not significant (p > 0.05). Error bars: SD.

To investigate a wider range of pathways impacted by hypoxia in a spatially-resolved manner, we analyzed the gene expression profiles of cells from micropattern regions under different oxygen levels in the device. Cells were extracted with laser capture microdissection (LCM) from pillar center (PC) and pillar edge (PE), which represent hypoxic and near-normoxic (0.12~0.14 mol/m^3^ by simulation) regions, respectively (Fig. 4A). Gene expression was compared in two ways: (1) both PC and PE regions in the hypoxia device were compared to their corresponding regions in the normoxic samples, and (2) PC was compared to PE in the same samples under the respective normoxic or hypoxic conditions. Gene targets were selected to include a diverse range of cellular functions, including cell proliferation (*MKI67*)^46^, apoptosis (*BNIP3*, *DDIT4*)^47,48^, glycolytic metabolism (*SLC2A1*, *CAIX*, *PGK1*)^45,47,48^, and migration/metastasis (*SNAI1*, *VIM*, *CXCR4*)^49,50^.

When normalized to their normoxic counterparts, cells in hypoxic PC regions have down-regulated proliferation and up-regulated expression of genes related to apoptosis, glycolysis, and migration/metastasis (blue bars, p < 0.05, Student’s *t*-test, Fig. 4B). In contrast, the same analysis shows no significant up- or down-regulation of the same set of genes in the PE regions (orange bars, p > 0.05, Student’s *t*-test, Fig. 4B). When we analyze the differential gene expression between PC and PE in the same samples, we discovered that the gene expression in the cells from the hypoxia device are spatially regulated by the oxygen gradient, with down-regulated proliferation and up-regulated markers in PC for apoptosis, glycolysis and migration/metastasis (except *SNAI1*) (orange bars, p < 0.05, Student’s *t*-test, Fig. 4C). Similarly, the same analysis shows that there is no spatially resolved differences in gene expression in normoxic samples (blue bars, p > 0.05, Student’s *t*-test, Fig. 4C).

### Spatially resolved drug response is observed in the device

**Figure 5:**
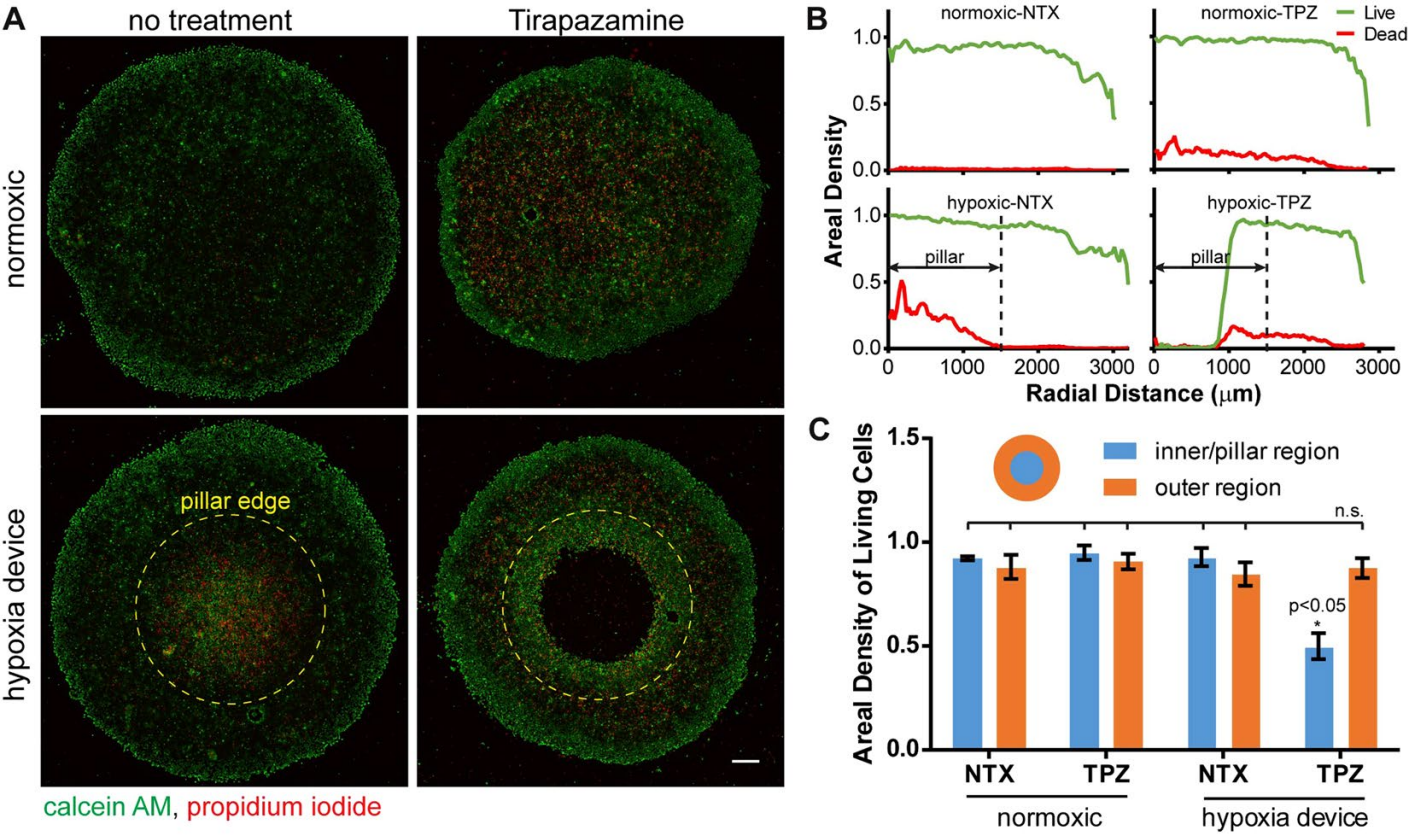
Cellular response to hypoxia-targeting drugs in hypoxia device. (**A**) Live-dead staining of micropatterned MCF-7 cells under normoxic condition or in hypoxia device, without treatment or under the treatment of tirapazamine (TPZ), a drug targeting hypoxic cells. Green: live (calcein); red: dead (propidium iodide). (**B**) Areal density of live (green) and dead (red) cells in the micropattern along the radial direction. (**C**) Proportion of live cells in the inner/pillar region versus outer region (N = 3). One-way ANOVA; n.s.: not significant (p > 0.05). Error bars: SD.

To assess the response of cancer cells to drug treatment under a hypoxic gradient, as well as the feasibility of our microdevice for drug screening assays, we performed cell viability assays with TPZ treatment in the microdevice. TPZ is an experimental anticancer prodrug that is 15- to 50-fold more selective at targeting hypoxic human cancer cells than their normoxic counterparts^51,52^. Cells were pre-conditioned with hypoxia in our microdevice for 12 hours, which was considered sufficient to induce cellular adaptations to hypoxia^47^, before being treated with TPZ for 24 hours. We used the TPZ concentration that inhibit 50% of the cancer cell growth (IC50)^53^ and differentially kill hypoxic cells over normoxic cells in the dosage test (Supplementary Fig. 1). Live-dead staining revealed cytotoxicity caused by TPZ in both normoxic and hypoxic samples indicated by positive nuclear propidium iodide (PI) staining (Fig. 5A, tirapazamine column, and corresponding radial distributions in Fig. 5B). Non-treated cells under deep hypoxia also showed pronounced cell death (Fig. 5A,B, lower left panel). Most strikingly, cells under severe hypoxia (corresponding to ≤ 0.03 mol/m^3^ oxygen level by COMSOL^®^ simulation) in the microdevice were eliminated by TPZ treatment (Fig. 5A,B, lower right panel). As dead cells can be washed off in the staining process, we chose the areal fraction of living cells with positive calcein staining as the readout for TPZ cytotoxicity. We quantified the live-cell fractions in the micropatterns inside and outside the 1.5 mm radius, which corresponds to the pillar radius. TPZ treatment caused significant reduction in the live-cell fraction only in the hypoxia microdevice, while all other conditions were not statistically different from each other (Fig. 5C, one-way Analysis of Variance, ANOVA).

## Discussion

In this report, we have introduced a novel microdevice platform to study tumor microenvironment under a hypoxic gradient. It can accurately generate and control oxygen gradients, eliminates complex microfluidic fabrication and handling, allows for incorporation of additional tumor components, and is compatible with high-content imaging-based analysis and high-throughput applications. These features have only been partially achieved by other individual platforms^24,27–33^. By combining cell-driven oxygen consumption and controlled passive oxygen diffusion, we eliminated the microfluidic components commonly used by others^21,54^, thus greatly simplifying the design of the microdevice and cell culture operations. The lateral oxygen gradient created on a monolayer cell culture also allows for real-time, high-content investigation of cellular phenotypes and behaviors with wide-field microscopy-based techniques, as demonstrated by the LCM-based gene expression analysis. This simplicity and compatibility will likely facilitate the adoption of our technique in biological research laboratories that usually lack engineering equipment or expertise to handle microfluidic devices, as well as in pharmaceutical industry that requires simplicity, scalability, and reproducibility^55,56^. In the present study, we only demonstrated the use of the platform for cell micropatterns larger than the pillar so that cells outside the pillar can be referred to as an internal normoxic control, and for up to 36 hours of cell culture (12 hours of conditioning and 24 hours of TPZ treatment), which is sufficient to induce gene and protein expressions as well as drug response. Notably, we have adapted the platform for a growing “tumor nest” culture by culturing cell micropatterns smaller than the pillar, which has a co-evolving hypoxic gradient with the growing cell island. We have also extended cell culture to 96 hours to capture additional hypoxic responses. Moreover, our platform can also be used beyond cancer to study other biological processes and cell types affected by hypoxia, such as the differentiation of embryonic stem cells^57^ and induced pluripotent stem cells (iPSCs)^58^, wound healing^59^, and immunoediting^60,61^.

Micromilling was used to fabricate the hypoxia microdevice in this study. The technique allows for materials with desired (low) oxygen permeability, which is not attainable with PDMS in conventional soft lithography^62^. Importantly, a unique strength of the device and fabrication is that the diffusion barrier pillar can be milled with flexible sizes (e.g. lateral or gap dimensions), arbitrary geometries (e.g. squares, ovals, or those mimicking real tumor shapes) and topologies (e.g. conical or spherical shapes) to alter the overall oxygen distribution in the gap. With the assistance of computer simulation, we can thus design oxygen distribution profiles in the microdevices to reflect the heterogeneous oxygen landscape in tumors with various sizes and cancer types, and at different stages^39,63^. As a rapid prototyping technique, micromilling also allows for quick iteration of design parameters. On the other hand, once the parameters are set for a given study or application, alternative fabrication approaches such as inject molding can be utilized to fabricate the microdevice in large scales^64^.

Another important feature of our tumor microdevice platform is in its ability to incorporate additional components and features of tumor microenvironment. For example, the collagen I coating can be replaced with other ECM types (e.g. collagen IV, fibronectin, hyaluronic acid, etc.) that play unique roles in cancer progression and therapeutic resistance^65,66^. The glass substrate can be supplemented with a layer of elastic material (e.g. acrylamide gel^67^ or PDMS micropillar array^68^) to understand the interplay of cellular mechanics with hypoxia. Additional cell types, such as immune cells and fibroblasts can be incorporated to reveal their crosstalk with cancer cells in an oxygen gradient^41^. While the present study is focused on 2D monolayer cultures, we are integrating scaffold biomaterials and bioprinting techniques to create thin-layer 3D cultures in the platform to further recapitulate cellular behaviors unique to 3D cultures^69^.

Finite element analysis through COMSOL Multiphysics^®^ was extensively used to simulate oxygen levels and distributions in the microdevice, to confirm the concept and adjust the design in the study. It is noteworthy that the fidelity of the simulation to reality is dependent on the physical parameters used in the model^70^. One of the key parameters is the oxygen consumption behavior of the cells. We used an oxygen consumption rate of MCF-7 cells reported by others^63^. For hypoxic conditions, we followed previous reports^70,71^ to assume that the cells have a Michaelis-Menten-type consumption rate depending on the actual oxygen levels (above a critical value), which drops to zero when oxygen level falls below the critical value^70^. To mimic a more realistic spatiotemporal oxygen profile, commercial assays (such as the Seahorse assay^72^) can be used to further validate or replace the concentration-dependent oxygen consumption equation for given cells of interest.

To complement the numerical simulation, we measured the oxygen distribution in the microdevice with microparticle-based oxygen sensors embedded under the pillar. It should be noted that the oxygen sensor particles showed highly variable fluorescent signals near the center of the pillar where deep hypoxia is induced by the cell micropattern (Fig. 2C, orange curve). There was also high variability of fluorescence intensity, calibration curves, and oxygen measurement from different devices (seen as high standard deviation, SD in Fig. 2D). Therefore, in its current form, the oxygen sensor layer method is still only a semi-quantitative analysis. The model also did not consider the photobleaching of the luminophore and the distribution of the ruthenium material in the silica and silicone phases of the sensor layer, which have been suggested to influence the linearity and sensitivity of the measurement^73^. In the future, we can improve the measurement by adopting a two-site oxygen binding model for the multi-phase sensor layer^40,73^. On the other hand, the fluorescence lifetime of the ruthenium-based oxygen sensors is also dependent on oxygen levels dictated by a similar Stern-Volmer model^40,73^. Since the lifetime of fluorescence is an intrinsic property of a fluorophore and independent of fluorescent intensity^74^, we will alternatively use fluorescence lifetime imaging microscopy (FLIM) to more accurately measure the oxygen levels^75^. It is important to note that a gradient of nutrients and soluble factors can be similarly induced by cellular metabolism and biological activities. We will further integrate chemical and optical sensors to measure other microenvironmental factors such as glucose^76^, cytokines^77^, metabolites^78^, and pH^79^.

Our microdevice can capture the spatial heterogeneity of cellular phenotypes induced by a hypoxic gradient. Molecules and proteins regulated by hypoxia can be immunostained and correlated with the oxygen gradient, as seen in the Hypoxyprobe-1^TM^ and Glut-1 staining (Fig. 3). As a proof-of-concept, we have also interfaced the platform with LCM, another microscopy-based technique, to analyze the spatial profile of gene expressions related to a wide range of biological behaviors (Fig. 4). With next-generation sequencing and proteomic technologies^80,81^, it will allow for transcriptome- and proteome-level analysis of the hypoxic tumor microenvironment on a single-cell level, and reveal signaling network and crosstalk linked to cancer progression and therapeutic response. These include, but are not limited to, cellular metabolism^82^, CSCs^83^, EMT^84^, radioresistance^82^, as well as biomarkers related to disease prognosis^45,82^.

We confirmed the cytotoxic effects of TPZ, an experimental drug that is preferentially activated in hypoxic environments^85^, on cancer cells experiencing a hypoxic gradient (Fig. 5). We observed a striking “death zone” near the center of the pillar under TPZ treatment, with a sharp boundary between the dead and live cell area. The result suggests the highly selective nature of TPZ treatment on cells below a hypoxic threshold. Notably, we observed increased cell deaths in untreated hypoxic samples (Fig. 5A,B, lower left panel) in agreement with the gene expression data that indicate enhanced apoptosis in the PC region against the PE region and the normoxic control (Fig. 4B,C). We also observed TPZ-induced cytotoxicity in the normoxic samples under the 50 µM TPZ treatment condition (Fig. 5A,B, upper right panel), which is consistent with the TPZ-mediated cell killing in normoxic cultures in the dose-response measurement (Supplementary Fig. 1). Interestingly, calcein signal in the live-dead staining was preferentially enhanced in the central hypoxic areas, at the edges of the “death zone”, and at the periphery of micropatterns. It may be attributed to reduced self-quenching of calcein dye in more extended cells^86^ as a result of increased growth areas due to micropattern edge effect or dead neighboring cells, or reduced expression of multidrug transporter^87^. To minimize the influence of calcein fluorescence intensity in our quantification, areal fraction of positive calcein staining, instead of the total intensity, was thus calculated for Fig. 5C. Future experiments on TPZ dosage and its relation to the radii of the “death zone” can further reveal the adaptability of the drug to different hypoxic levels. We can also use our microdevice as a drug testing/screening platform to assess the efficacy of combinatorial treatments with chemo-, targeted- and immuno-therapeutic drugs to eradicate heterogeneous cancer populations in the hypoxic tumor^88^, to accelerate the discovery of more effective cancer drug regimens.

In summary, we present a tumor microdevice platform that recapitulates the hypoxic gradient in tumor microenvironment for high-content and high-throughput applications. We demonstrated the establishment of the oxygen profile through multiphysics simulation, optical sensor measurement, immunostaining, spatially-resolved gene expression analysis, and hypoxia-targeted drug treatment. It is compatible with high content imaging, live-cell tracking, and single-cell analyses. It is also adaptable with additional microenvironmental components and biosensors. Our flexible and scalable platform will allow for extensive investigation of tumor biology and other hypoxia-related biosystems, and also serve as a powerful tool for therapeutic discoveries.

## Materials and Methods

### Cell culture and micropatterning

MCF-7 human breast cancer cells were purchased from ATCC and maintained in Dulbecco’s Minimum Essential Medium (DMEM; Thermo Fisher) supplemented with 10% fetal bovine serum (FBS; Omega Scientific), 100 U mL^−1^ penicillin, and 100 μg mL^−1^ streptomycin (Thermo Fisher), in a humidified incubator maintained at 37 °C and 5% CO_2_. Round glass coverslips (12 mm in diameter; Fisher Scientific) were immersed in hot commercial detergent, rinsed with deionized water, and dried with air. The coverslips were then treated with plasma (Harrick Plasma, Model PDC-001-HP) and silanized with 1% aminopropyltriathoxysilane (Fisher Scientific) for 15 minutes. Upon extensive rinsing, coverslips were dried with air and cured at 100 °C for 1 hour. Next, silanized coverslips were coated with 0.1 mg mL^−1^ rat tail collagen type I (Corning) in 4 °C for 3 hours under shaking conditions. Micropattern designs were modeled in CorelDrawX7 (Corel Corporation) and fabricated into 250 μm thick PDMS stencils (Rogers Corporation) by a laser engraver (Epilog). Stencil design was a circular feature of 5 mm diameter cut into a 13 mm circle. Stencils were then thoroughly rinsed in 70% isopropanol and deionized water, air-dried, and aligned onto the collagen-coated coverslips. The whole substrate was blocked with 0.2% w/v pluronic F-127 (Sigma) diluted in 1x PBS, rinsed with PBS, then with DMEM. Next, 300,000 MCF-7 human breast cancer cells (ATCC) were seeded. After cells adhered, PDMS stencils were peeled off and the glass coverslips with micropatterned cancer cells were briefly rinsed^41^.

### Fabrication of hypoxia device

The design and toolpaths for the hypoxia microdevice was created using Autodesk Fusion360 (Autodesk, Inc.). The design consists of a base structure to immobilize the coverslip and a cap structure with a diffusion barrier pillar. Subsequently, the design was converted into a g-code, imported into a commercial software (Otherplan, Other Machine Co.), and milled with a computer numerical control machine (Othermill V2, Other Machine Co.). The base and cap structures were milled in Delrin® and polycarbonate, respectively. Upon mechanical polishing using 1000 grit sandpaper (3 M), microdevices were autoclaved before use. For drug assays, the polycarbonate cap was vapor polished with methylene chloride inside a fume hood to achieve optical transparency^34^.

Substrates with micropatterned cells were set into autoclaved microdevices and incubated for specified times. Samples were then fixed in 4% paraformaldehyde (PFA; Electron Microscopy Sciences) for 10 minutes or ice-cold 100% ethanol for immunostaining and LCM, respectively.

### COMSOL Multiphysics^®^ modeling

The transient diffusion of oxygen in our microdevice was modeled using finite element methods (COMSOL Multiphysics^®^ software, COMSOL Inc.). Passive oxygen diffusion within the media was assumed to be governed by the generic diffusion equation of gas in water^70^, with a diffusion coefficient of 3 × 10^−9^ m^2^ s^−1^. Boundary conditions were approximated so that the microdevice was impermeable to oxygen and the media surface in direct contact with atmospheric media had a fixed concentration of oxygen corresponding to normoxic levels (0.2 mol m^−3^). Cellular oxygen consumption was assumed to follow Michaelis-Menten kinetics with a logistic function constraining consumption below a critical oxygen level:

1 $${R}_{{O}_{2}}={R}_{{\max }}(\frac{c}{c+{k}_{MM,{O}_{2}}})\cdot \delta (C > {C}_{cr})$$

where *R*_*max*_ is the maximum oxygen consumption rate of MCF-7 cells adjusted for their average cell volume (0.034 mol s^−1^ m^−3^)^63,70,89^, ${k}_{MM,{O}_{2}}$ is the Michaelis-Menten constant corresponding to the oxygen concentration where consumption is half maximal, *C*_*cr*_ is the critical oxygen concentration below which necrosis is assumed to happen and cells cease oxygen consumption, and δ is the step-down function accounting for the termination of oxygen consumption^70^. The step-down function was COMSOL’s smoothed Heaviside function with a continuous first derivative and no overshoot (flc1hs in COMSOL Multiphysics^®^). All geometries in the model were defined with an extremely fine mesh in COMSOL Multiphysics. The model was then solved as a time-dependent study up to 1,440 minutes (time step = 1 minute), where the device and the media were assumed to be equilibrated to normoxia at t = 0.

### Physiological oxygen concentration measurement

Oxygen levels were measured using fluorophore-based microparticle sensors^40^. Briefly, 2 g of 10–14 μm grade 7 silica gel (Sigma Aldrich) were stirred with 40 mL of 0.1 N NaOH for 30 minutes; then with 10 mL ethanol solutions of 0.5 mM tris(4,7-diphenyl-1,10-phenanthroline) ruthenium(II) dichloride (Thermo Fisher) and 0.5 mM Nile blue chloride (Sigma Aldrich), respectively, for 30 minutes. The solution was then centrifuged for 20 minutes at 1900 × g. The pellet was washed and centrifuged with the same settings thrice with deionized water, and once with ethanol. The fluorophore-immobilized silica gel pellet was then dried in a 70 °C oven overnight. Simultaneously, a lid structure that fits the diffusion barrier pillar was milled with polycarbonate and silanized with trichloro(1 H, 1 H,2 H,2H-perfluorooctyle)silane (Sigma Aldrich) overnight. The following day, fluorophore-immobilized silica gel was mixed with PDMS of 1:10 base to curing agent (Sylgard 184 elastomer kit; Dow Corning) at a 1:20 ratio in an AR-100 Thinky mixer (Thinky U.S.A., Inc.). The mixture was then poured onto the microdevice’s pillar, covered with the lid, and cured overnight. Upon detaching the lid, the coated cap was imaged in 1x PBS equilibrated with normoxic air and then incubated with micropatterned cells. After 24 hours, fluorescence from the pillar surface was imaged.

### Immunostaining

After 24 hours of hypoxia or normoxia incubation, 4% PFA-fixed samples were permeabilized with 0.1% Triton X-100 (Fisher Scientific), blocked with 4% bovine serum albumin (GE Healthcare Bio-Sciences), incubated in primary and secondary antibody, and mounted with FluoroGel II containing DAPI (Electron Microscopy Sciences) onto glass slides. Primary antibodies used were monoclonal anti-pimonidazole antibody (9.7.11, 1:50) (Hypoxyprobe, Inc.) and anti-Glucose Transporter 1 (Glut-1) antibody (ab15309, 1:200) (Abcam). In the case of pimonidazole staining, cells were incubated with 200 μM pimonidazole 2 hours before fixation. Pimonidazole and Glut-1 were detected with Alexa Fluor fluorescent dye-conjugated secondary antibodies (Life Technologies). A Nikon inverted fluorescent microscope was used to image immunostained samples.

### Gene expression assay

Additionally, cells were laser capture microdissected (Arcturus XT Laser Capture Microdissection System) at locations corresponding to the pillar center and pillar edge after 24 hours of hypoxia or normoxia treatment. RNA was extracted from these cells (Arcturus PicoPure RNA Isolation Kit) and the quality was evaluated with a Varioskan LUX multimode microplate reader (Thermo Fisher Scientific). RNA samples were then reverse transcribed into cDNA with a T100^TM^ Thermal Cycler (BIO-RAD) and amplified with the T100 CFX384 Touch Real-Time PCR Detection System (BIO-RAD) to assess expression of selected gene candidates^45,47–50,90–92^. Data were normalized against β-actin, a housekeeping gene that was confirmed to have relatively stable expression regardless of normoxic or hypoxic conditions^93^, and an internal sample control (ΔΔCt method). These ΔΔCt values were plotted in log2 scale and used to assess gene expression control.

### Hypoxia-activated drug assays

Micropatterned cells were pre-conditioned in normoxic conditions (no microdevice) or hypoxic conditions (microdevice) for 12 hours. Next, media was replaced with 50 μM TPZ (Sigma Aldrich), a hypoxia-activated anticancer prodrug, for 24 hours. Cells were rinsed with fresh media and stained for calcein-AM (Sigma Aldrich) and propidium iodide (PI) (Thermo Fisher Scientific) for 30 minutes at room temperature. Cell survival was quantified by the fraction of cells expressing positive calcein signal.

### Image analysis

Images were analyzed using the ImageJ and MATLAB software. For oxygen measurements, fluorescent intensity from identified sensor microparticles was quantified independently in each fluorophore’s corresponding fluorescence channel (Acridine Orange for ruthenium compound and Cy5 for Nile blue chloride). Raw, pixel-by-pixel fluorescence from tris(4,7-diphenyl-1,10-phenanthroline) ruthenium(II) dichloride was divided by those from Nile blue chloride to obtain a ratio of differential quenching in the oxygen-sensitive and -insensitive fluorophores depending on oxygen levels. This data was then binned into concentric circles with fixed step size (13.5 µm) from the measured centroid of each pillar and related to “sensed” oxygen concentration following a conventional Stern-Volmer model^40,73^:

2 $$\frac{{I}_{R,O}}{{I}_{R}}-1={K}_{SV[{O}_{2}]}$$

where *I*_*R*_,_*O*_ and *I*_*R*_ are the fluorescence ratio of the two fluorophores in the absence and presence of oxygen, respectively, and *K*_*SV*_ is the Stern-Volmer quenching constant. Derived oxygen concentrations for each bin were plotted against pillar radii.

For immunostained samples, the fraction of micropattern area with fluorescence above a pre-defined threshold value was measured. This fraction was also binned into 100 radially evolving concentric circles and plotted against micropattern radii.

For the drug assay, the fraction of calcein positive cells (live cells) within (corresponding to hypoxia-induced cells under the pillar) and outside (corresponding to near-normoxic cells outside the pillar) the 1.5 mm radius was quantified. The fraction of PI positive cells (dead cells) was also quantified. The respective fractions were plotted against micropattern radii, similarly to previous image analyses. All data are plotted using Prism (GraphPad Software, Inc.).

### Statistical analysis

All data are presented in mean ± S.D. Pearson’s correlation coefficient (r) was used to depict correlation between readings from the oxygen sensors and the COMSOL simulation, as well as pimonidazole and Glut-1 staining. Statistics for gene expression was generated using Student’s t-test. Statistics for drug treatment study was assessed using the one-way ANOVA. In all statistical analysis, p < 0.05 was considered significant.

## Electronic supplementary material

Supplementary Information
