# Supplementary material for: A Microdevice Platform Recapitulating Hypoxic Tumor Microenvironments
Source: Sci Rep. 2017 Nov 9;7:15233. doi: 10.1038/s41598-017-15583-3 (PMC5680268; doi:10.1038/s41598-017-15583-3)
Supplement: Supplementary file 1 — Supplementary Information [file 41598_2017_15583_MOESM1_ESM.pdf]

Supplementary Information

A Microdevice Platform Recapitulating Hypoxic Tumor Microenvironments

Yuta Ando[1], Hoang P. Ta[1], Daniel P. Yen[1], Sang-Sin Lee[1], Sneha Raola[1], Keyue Shen[1,2,3]

[1]Department of Biomedical Engineering, Viterbi School of Engineering, University of Southern California, Los Angeles, CA 90089

[2]Norris Comprehensive Cancer Center, Keck School of Medicine, University of Southern California, Los Angeles, CA 90033

[3]Department of Stem Cell Biology and Regenerative Medicine, Keck School of Medicine, University of Southern California, Los Angeles, CA 90033

Correspondence should be addressed to Keyue Shen (keyue.shen@usc.edu)

Supplementary Figures

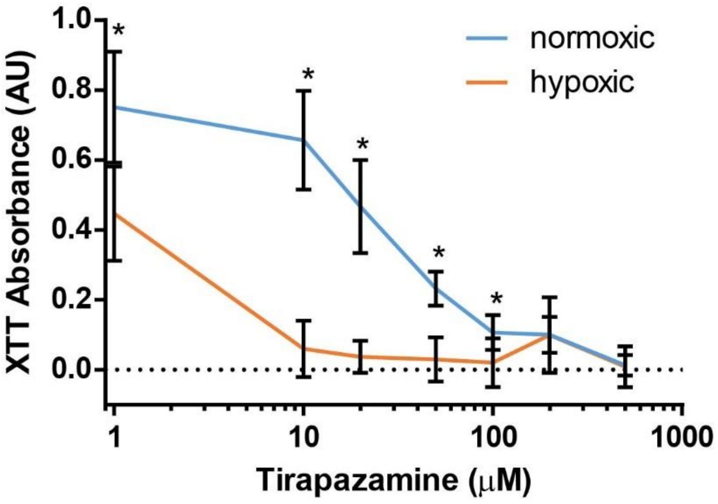

Supplementary Figure 1. Dose response of MCF-7 cells to tirapazamine (TPZ) under normoxic and hypoxic conditions. 10,000 MCF-7 cells were seeded in a 96-well plate. Cells were pre-conditioned inside a cell culture incubator (normoxic) or inside a hypoxia chamber (hypoxic, 1% oxygen) for 12 hours before treated with different doses of TPZ for 24 hours. XTT cell proliferation assay was carried out according to the manufacturer's protocol indicative of metabolic activities of the surviving cells. *: $p<0.05$ between normoxic and hypoxic conditions, Student's t-test.
